# Supplementary material for: Relationship between Metabolomics Profile of Perilymph in Cochlear-Implanted Patients and Duration of Hearing Loss
Source: Metabolites. 2019 Nov 1;9(11):262. doi: 10.3390/metabo9110262 (PMC6918144; doi:10.3390/metabo9110262)
Supplement: Supplementary file 1 [file metabolites-09-00262-s001.pdf]

Supplementary material : 106 metabolites identified

Metabolite	Chemical class	HMDB
1-OLEOYL-RAC-GLYCEROL	Monoradylgycerol	HMDB0011567
3-METHOXY-L-TYROSINE	Amino acids, peptides, and analogues	HMDB0001434
5-OXO-L-PROLINE	Carboxylic acids	HMDB0000267
ADENOSINE	Purine nucleoside	HMDB0000050
CITRULLINE	Carboxylic acids	HMDB0000904
CORTISOL	Hydroxysteroids	HMDB0000063
CREATINE	Carboxylic acids	HMDB0000064
CREATININE	Amino acids, peptides and analogues	HMDB0000562
DL-5-HYDROXYLYSINE	Amino acids, peptides, and analogues	HMDB0000450
ETHANOLAMINE	Amines	HMDB0000149
HYPOXANTHINE	Purines and purine derivatives	HMDB0000157
L-CARNITINE	Quaternary ammonium salts	HMDB0000062
L-HISTIDINE	Amino acids, peptides and analogues	HMDB0000177
L-SERINE	Amino acids, peptides and analogues	HMDB0000187
N-ACETYL-L-LEUCINE	Carboxylic acids	HMDB0011756
N6, N6, N6-TRIMETHYL-L-LYSINE	Amino acids, peptides, and analogues	HMDB0001325
NICOTINAMIDE	Pyridinecarboxylic acids and derivatives	HMDB0001406
O-ACETYL-L-CARNITINE	Fatty acids esters	HMDB0000201
SN-GLYCERO-3-PHOSPHOCHOLINE	Glycerophosphocholines	HMDB0000086
TRIGONELLINE	Alkaloids and derivatives	HMDB0000875
2-HYDROXYPHENYLACETIC ACID	Benzene and substitutes derivatives	HMDB0000669
2-METHYLPROPANOATE	Carboxylic acids and derivatives	HMDB0001873
3-AMINO-4-HYDROXYBENZOIC ACID	Benzoic acids and derivatives	HMDB0001476
3-HYDROXY-3-METHYLGLUTARATE	Fatty acyls	HMDB0000355
3-HYDROXYANTHRANILATE	Benzene and substitutes derivatives	HMDB0001476
3-HYDROXYBUTANOIC ACID	Beta hydroxy acids and derivatives	HMDB0000357
3-METHYLGLUTARIC ACID	Fatty acids and conjugates	HMDB0000752
PIMELIC ACID	Fatty acids and conjugates	HMDB0000857
ADIPIC ACID	Fatty acids and conjugates	HMDB0000448
ALPHA-D-GLUCOSE	Carbohydrates and carbohydrate conjugates	HMDB0003345
ALPHA-HYDROXYISOBUTYRIC ACID	Hydroxy acids and derivatives	HMDB0000729
BETA-ALANINE	Amino acids, peptides and analogues	HMDB0000056
BUTANOATE	Fatty acyls	HMDB0000039
D-PANTOTHENIC ACID	Carboxylic acids and derivatives	HMDB0000210
D-RIBOSE-5-PHOSPHATE	Organooxygen compounds	HMDB0001548
GLUTARIC ACID	Carboxylic acids and derivatives	HMDB0000661
L-ARGININE	Amino acids, peptides and analogues	HMDB0000517

L-ASPARAGINE	Carboxylic acids and derivatives	HMDB0000168
MANNITOL	Carbohydrates and carbohydrate conjugates	HMDB0000765
MONO-ETHYL MALONATE	Carboxylic acids and derivatives	HMDB0000576
PENTANOATE	Fatty acids and conjugates	HMDB0000892
S)-LACTATE	Hydroxy acids and derivatives	HMDB0000190
S.S)-TARTARIC ACID	Carbohydrates and carbohydrate conjugates	HMDB0029878
SUBERIC ACID	Fatty acids and conjugates	HMDB0000893
XANTHINE	Imidazopyrimidines	HMDB0000292
10-HYDROXYDECANOATE	Medium-chain hydroxy acids and derivatives	HMDB0002203
1-AMINOCYCLOPROPANE-1-CARBOXYLATE	Carboxylic acids and derivatives	HMDB0036458
2-DEOXY-D-GLUCOSE	Fatty alcohols	HMDB0062477
GABA	Carboxylic acids and derivatives	HMDB0000112
4-GUANIDINOBUTANOATE	Carboxylic acids and derivatives	HMDB0003464
4-HYDROXY-L-PROLINE	Amino acids, peptides and analogues	HMDB0000725
ALPHA-AMINOADIPATE	Carboxylic acids and derivatives	HMDB0000510
L-ALANINE	Amino acids, peptides and analogues	HMDB0000161
DEOXYCARNITINE	Fatty acids and conjugates	HMDB0001161
DIETHANOLAMINE	Amines	HMDB0004437
L-MANNOSAMINE	Carbohydrates and carbohydrate conjugates	HMDB0000230
GLYCINE	Amino acids, peptides and analogues	HMDB0000123
GUANIDINOACETATE	Amino acids, peptides and analogues	HMDB0000128
HOMOSERINE	Carboxylic acids and derivatives	HMDB0000719
L-ANSERINE	Peptidomimetics	HMDB0000194
L-GLUTAMINE	Carboxylic acids	HMDB0000641
LL-2,6-DIAMINOHEPTANEDIOATE	Carboxylic acids and derivatives	HMDB0001370
L-LYSINE	Amino acids, peptides and analogues	HMDB0000182
L-METHIONINE	Carboxylic acids and derivatives	HMDB0000696
L-PHENYLALANINE	Carboxylic acids and derivatives	HMDB0000159
L-PROLINE	Amino acids, peptides and analogues	HMDB0000162
L-THREONINE	Carboxylic acids and derivatives	HMDB0000167
L-VALINE	Amino acids, peptides and analogues	HMDB0000883
N(PAI)-METHYL-L-HISTIDINE	Carboxylic acids and derivatives	HMDB0000479
N-ACETYL-L-ALANINE	Carboxylic acids and derivatives	HMDB0000766
N-ACETYLPUTRESCINE	Carboximidic acids and derivatives	HMDB0002064
CN,N,N TRIMETHYL LYSINE	Amino acids, peptides, and analogues	HMDB0001325
OMEGA-HYDROXYDODECANOIC ACID	Hydroxy acids and derivatives	HMDB0002059
PARAXANTHINE	Imidazopyrimidines	HMDB0001860
PHOSPHOCHOLINE	Organonitrogen compounds	HMDB0001565
RAC-GLYCEROL-1-MYRISTATE	Monoradylglycerol	PubChem CID 79050
SEROTONIN	Tryptamines and derivatives	HMDB0000259

SORBATE	Carbohydrates and carbohydrate conjugates	HMDB0000247
URACIL	Pyrimidines ans pyrimidine derivatives	HMDB0000300
2-HYDROXYBUTYRIC ACID	Alpha hydroxy acids and derivatives	HMDB0000008
3-(4-HYDROXYPHENYL)LACTATE	Phenylpropanoic acids	HMDB0000755
3-UREIDOPROPIONATE	Organic carbonic acids and derivatives	HMDB0000026
4-IMIDAZOLEACETIC_ACID	Azoles	HMDB0002024
4-METHYL-2-OXO-PENTANOIC ACID	Short-chain keto acids and derivatives	HMDB0000695
4-METHYL-2-OXOVALERIC ACID	Short-chain keto acids and derivatives	HMDB0000695
5-OXO-D-PROLINE	Amino acids, peptides and analogues	HMDB0000805
ALLANTOIN	Imidazoles	HMDB0000462
ALPHA-D-GALACTOSE-1-PHOSPHATE	Carbohydrates and carbohydrate conjugates	HMDB0000645
CIS-4-HYDROXY-D-PROLINE	Carboxylic acids and derivatives	HMDB0060460
D-GLUCONATE	Carbohydrates and carbohydrate conjugates	HMDB0000625
D-GLUCURONIC ACID	Organooxygen compounds	HMDB0000127
D-LACTOSE	Carbohydrates and carbohydrate conjugates	HMDB0000825
D-RIBOSE	Carbohydrates and carbohydrate conjugates	HMDB0000283
ETHANOLAMINE PHOSPHATE	Organic phosphoric acids and derivatives	HMDB0000224
GALACTITOL	Carbohydrates and carbohydrate conjugates	HMDB0000107
INOSINE	Purine nucleoside	HMDB0000195
L-ALLOTHREONINE	Carboxylic acids and derivatives	HMDB0004041
L-ARABITOL	Organooxygen compounds	HMDB0001851
L-CYSTINE	Carboxylic acids and derivatives	HMDB0000192
MALTOSE	Organooxygen compounds	HMDB0000163
METHYL JASMONATE	Fatty acyls	HMDB0036583
METHYLMALONATE	Carboxylic acids and derivatives	HMDB0000202
N-ACETYLNEURAMINATE	Organooxygen compounds	HMDB0000230
PANTOLACTONE	Gamma butyrolactones	HMDB0059876
SUCCINATE	Dicarboxylic acids and derivatives	HMDB0000254
TAURINE	Organosulfonic acids and derivatives	HMDB0000251
THEOPHYLLINE	Imidazopyrimidines	HMDB0001889
